# Evaluation of CINA® LVO artificial intelligence software for detection of large vessel occlusion in brain CT angiography

**DOI:** 10.1016/j.ejro.2023.100542

**Published:** 2023-12-15

**Authors:** Helena Mellander, Amir Hillal, Teresa Ullberg, Johan Wassélius

**Affiliations:** a Diagnostic Radiology, Department of Neuroradiology and Odontology, Center for Medical Imaging and Physiology, Skåne University Hospital, Lund, Sweden; b Department of Clinical Sciences, Lund University, Lund, Sweden

**Keywords:** Acute Ischemic stroke, Computed Tomography Angiography, Artificial Intelligence, Diagnostic Tests, Software Validation

## Abstract

**Objective:**

To systematically evaluate the ability of the CINA® LVO software to detect large vessel occlusions eligible for mechanical thrombectomy on CTA using conventional neuroradiological assessment as gold standard.

**Methods:**

Retrospectively, two hundred consecutive patients referred for a brain CTA and two hundred patients that had been subject for endovascular thrombectomy, with an accessible preceding CTA, were assessed for large vessel occlusions (LVO) using the CINA® LVO software. The patients were sub-grouped by occlusion site. The original radiology report was used as ground truth and cases with disagreement were reassessed. Two-by-two tables were created and measures for LVO detection were calculated.

**Results:**

A total of four-hundred patients were included; 221 LVOs were present in 215 patients (54 %). The overall specificity was high for LVOs in the anterior circulation (93 %). The overall sensitivity for LVOs in the anterior circulation was 54 % with the highest sensitivity for the M1 segment of the middle cerebral artery (87 %) and T-type internal carotid occlusions (84 %). The sensitivity was low for occlusions in the M2 segment of the middle cerebral artery (13 % and 0 % for proximal and distal M2 occlusions respectively) and in posterior circulation occlusions (0 %, not included in the intended use of the software).

**Conclusions:**

LVO detection sensitivity for the CINA® LVO software differs largely depending on the location of the occlusion, with low sensitivity for detection of some LVOs potentially eligible for mechanical thrombectomy. Further development of the software to increase sensitivity to all LVO locations would increase the clinical usefulness.

## Highlights

• The specificity in LVO detection for the CINA® LVO software is high.
• The CINA® LVO sensitivity for distal ICA and M2 occlusions is low.
• Thorough manual evaluation is still necessary if using the CINA® LVO software.

## 1 Introduction

Artificial intelligence (AI) is being increasingly explored for diagnostic applications in neuroradiology [1], [2]. AI software has so far been developed to detect intracranial aneurysms, calculate acute infarct- and penumbra volumes, and detect intracranial hemorrhage [3], [4].

Identification of Large Vessel Occlusions (LVO) on brain Computed Tomography Angiography (CTA) in acute stroke patients is an area where an AI algorithm potentially could be a valuable tool to decrease the time to diagnosis. Relevant occlusions to identify are primarily the ones targeted by mechanical thrombectomy, which include occlusion of the intracranial segments of the internal carotid artery (ICA), the intracranial segments of vertebral arteries (VA), the proximal segments of the middle (M1 and M2), anterior (A1) and posterior (P1) cerebral arteries (MCA, ACA and PCA) and the basilar artery (BA) [5], [6]. Fast and correct reading is fundamental for patient selection to mechanical thrombectomy, since every minute saved translates into better outcome for the patient [7], [8].

There are currently several available software for detection of LVOs, such as Viz LVO (Viz.ai Inc.), eCTA (Brainomix), and Rapid CTA (iSchemaView Inc.) [9] that integrate within the regular workflow and the Picture Archiving and Communication System (PACS). Canon now provides a comprehensive AI software package (^AUTO^Stroke, Canon Medical Systems) for assessment of intracranial hemorrhage, brain perfusion and LVO detection. The LVO detection software, CINA® LVO (Avicenna.AI), used in Canons ^AUTO^Stroke platform is based on deep learning, a subtype of machine learning based on artificial neural networks [3], and trained to detect and localize proximal LVOs in the anterior circulation (distal ICA, M1-occlusions and proximal M2-occlusions). The CINA® LVO algorithm was trained on 566 LVO cases, all examined during 2018 and included from a single center in the United States of America [10]. Initial clinical evaluation of the CINA® LVO software in patients presenting with acute stroke symptoms has resulted in an overall sensitivity of 73–98 % and a specificity of 98–98 % in recent studies [10], [11], [12]. These kinds of metrics are however only valid within the specific study population, and therefore needs to be confirmed in other populations, including high-incidence populations. To compose such an LVO-enriched population while minimizing selection bias, we combined consecutive CTA examinations (performed on any indication) with an equal number of consecutive CTA examinations in mechanical thrombectomy patients treated at our institution for LVO in the distal ICA (T-type and I-Type), the MCA (M1 and M2 segments) and A1-segment, as well as the Basilar artery and the proximal PCA (P1-segment) in the posterior circulation.

The aim of this study was to systematically evaluate the ability of the CINA® LVO software, included in the Canon ^AUTO^Stroke software package, to detect large vessel occlusions eligible for mechanical thrombectomy on CTA using conventional neuroradiological assessment as gold standard.

## 2 Materials and methods

### 2.1 Study population

We performed a retrospective search in our PACS (Picture and Archiving Communication System) to include 200 consecutive patients who had been referred for CTA of the cerebral arteries for any indication at any hospital within our region (a catchment area of 1.5 million people) prior to May 27th 2021. We then performed an additional search on May 31st 2021, and retrospectively included 200 consecutive patients who had been treated with endovascular thrombectomy (EVT) prior to May 31st 2021 in order to retrieve two cohorts that together could be considered an enriched LVO population. Each patient could only be included in one of the groups. For the patients of the CTA group, the urgency of the exam was noted (‘acute’, ‘sub-acute’ (< 1 week) or ‘not acute’) as well as the indication for the exam (‘fast track stroke’, ‘transient neurological deficits’, ‘persistent neurological deficits’, ‘headache’, ‘confusion’, ‘trauma’ and ‘other/multiple’).

Patients under the age of 18 years were excluded. For patients who underwent more than one examination within the time period, only the first was included in this study.

### 2.2 Data acquisition

Thin slice axial plane series were selected and analyzed by the ^AUTO^Stroke CINA® LVO software which was installed within the hospital’s IT environment and integrated with our PACS. For patients in the EVT group, the CT angiography directly prior to the endovascular treatment was used.

**Fig. 1:**
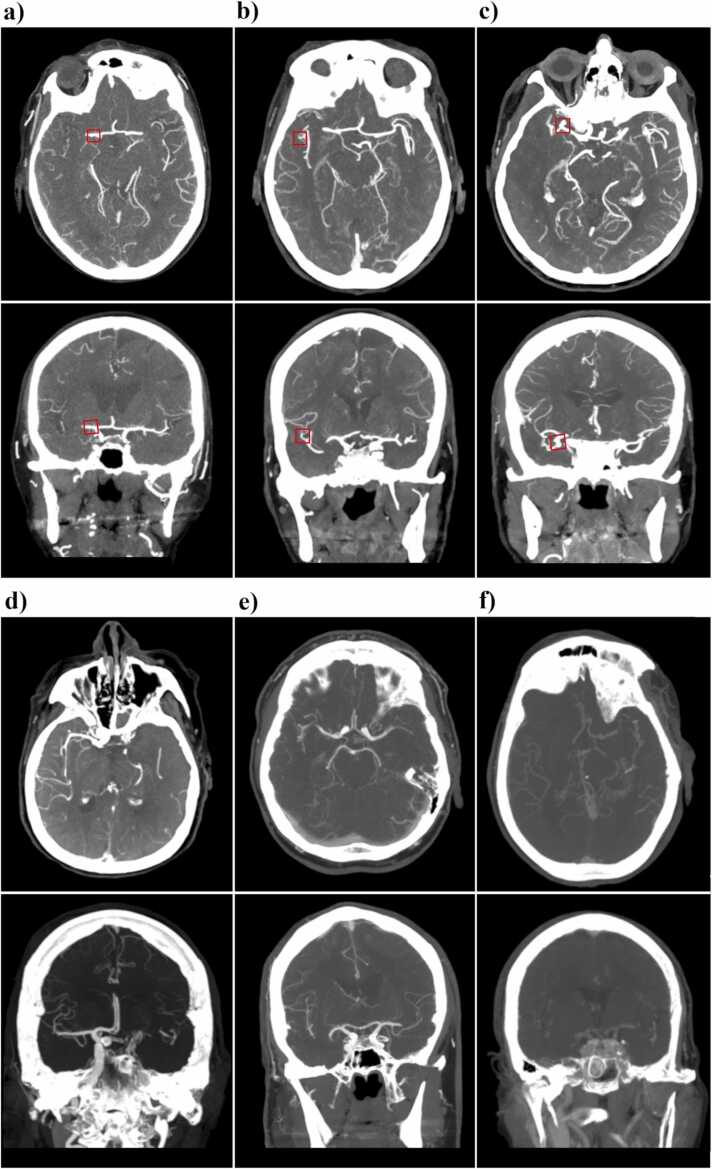
Examples with axial and coronal plane images for each case of a) true positive in a patient with a right-sided M1 occlusion, the red box placed by CINA® LVO to mark the detected occlusion, b) a case in which the software seems to have falsely interpreted a sharp curve of an M2 branch on the right side as an occlusion resulting in a false positive, c) another false positive in which an aneurysm in the right M1-bifurcation was interpreted as a LVO, d) a false negative case in a patient with a T-type ICA occlusion on the left side, undetected by the software, e) a false negative case in a patient with a M1 occlusion on the left side, undetected by CINA® LVO possibly due to interference by an adjacent vein, and f) a false negative due to an undetected M1 occulsion on the left side. LVO = large vessel occlusion; ICA = internal carotid artery.

The results from the ^AUTO^Stroke CINA® LVO software analysis were returned as images to the PACS, and all suspected LVOs detected by the software were indicated by a square mark in axial and coronal plane images (examples shown in Fig. 1).

**Fig. 2:**
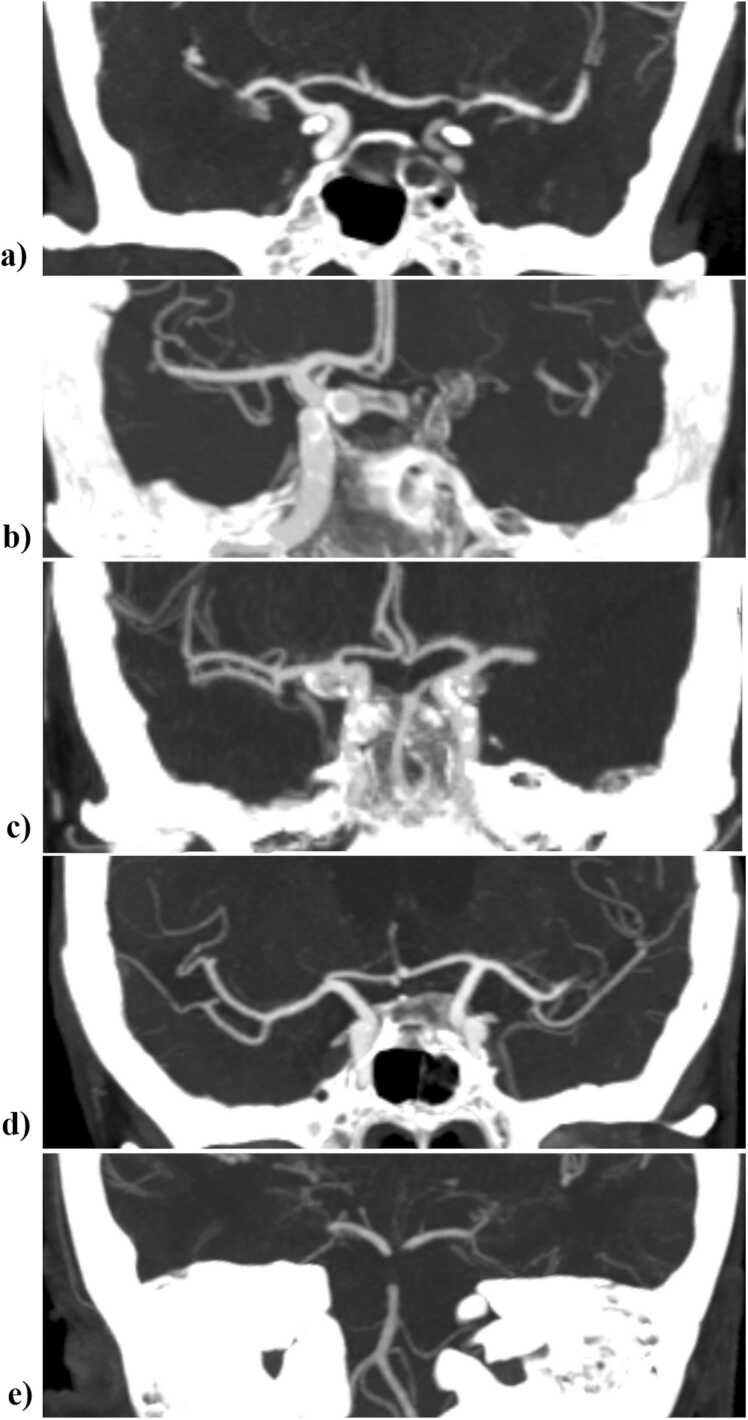
Examples of LVO locations; a) ICA I-type, b) ICA T-type, c) M1, d) proximal M2 and e) Basilar artery. LVO = Large vessel occlusion; ICA = internal carotid artery; M1 = first segment of middle cerebral artery; M2 = post bifurcation segments of the middle cerebral artery.

The original image reports for all patients were based on double review by two neuroradiologists or one radiologist and one neuroradiologist, in line with the clinical routine at our center. For this study, the presence of an LVO (yes/no), the side (left/right) and the site of occlusion (intracranial ICA (I-type, distal of the ophthalmic artery) or terminal ICA (T-type), M1-segment, the proximal half of the M2-segment (proximal M2), the distal half of the M2-segment (distal M2), BA, other or multiple) in the original radiological report, as well as in the ^AUTO^Stroke CINA® LVO report was noted (see Fig. 2 for examples of occlusion sites). The M2 segment was defined as the branches after the first bifurcation of the M1 segment beyond the lenticulostriate arteries and the antero-temporal branch.

The original manual reader – or in cases with disagreement – the result of the manual revaluation, was considered ground truth. For each case, the ^AUTO^Stroke CINA® LVO software report was assessed as true positive (when an LVO was correctly detected and the occlusion site correctly corresponding to that of the ground truth), false positive (when an suspected LVO was marked by the software but did not correspond to any finding in ground truth), true negative (when neither the software nor the ground truth detected an LVO) or false negative (when the ground truth assessed an LVO to be present but the software did not).

Any disagreements between the ^AUTO^Stroke CINA® LVO report and the original reports were resolved by manual reevaluation by a senior interventional neuroradiologist (with access to axial thin slice images as well as coronal and sagital image reconstructions) for the purpose of this study. For the false positive results, the images were assessed regarding any potential cause for softwares’ misinterpretation.

To further evaluate the performance stability of the software 30 exams from the CTA group and 30 exams from the EVT group were sent, on a separate time point, for repeated analysis by the ^AUTO^Stroke CINA and the results were noted separately.

### 2.3 Statistical analysis

IBM SPSS Statistics for Windows, version 25.0 (IBM Corp.) was used to calculate descriptive measures. Continuous data were presented as mean ± SD, ordinal and nominal data as n (%). An independent samples t-test was used to compare mean age between the EVT and CTA group, a Chi-Square test was used to compare the distribution of gender between the groups. MedCalc® Statistical Software version 20.009 (MedCalc Software Ltd) was used to calculate measures of diagnostic accuracy. Two-by-two tables were created to calculate diagnostic measures (sensitivity, specificity, positive and negative predictive values, and accuracy) for the CINA® LVO software. Subgroups were created to test the diagnostic ability for the CINA® LVO detection ability based on different occlusion sites, gender and age. Each occlusion was considered as one observation, patients with >1 occlusions could therefore be included in more than one subgroup.

## 3 Results

### 3.1 Patient and exam characteristics

Four hundred CTA examinations from 200 unselected patients and 200 pre-EVT patients were analyzed. There was a significant difference in mean age between the CTA and EVT groups, however the difference was small. There was no significant difference in distribution of gender between the groups. See Table 1 for details of patient characteristics. In the CTA group 169 (85 %) of the exams were acute and 54 of those were performed as ‘fast track stroke’, see Supplemental table A.1 for details of urgency and indication for the exam for the patients in the CTA group. A total of 221 LVOs were present in 215 patients (54 % of all, six patients had two separate LVOs). The most common occlusion site (according to ground truth) was M1 accounting for almost 40 % of all occlusions, the second most common occlusion site was M2 (28 % including both distal and proximal M2 occlusions).

**Table 1:** Patient characteristics and summary of occlusion sites.

	All patients (n = 400)	CTA patients (n = 200)	EVT patients (n = 200)	Sig. (p-value)
**Sex (women/men – n (%))**	203/197 (51/49)	103/97 (52/48)	100/100 (50/50)	0.764
**Age (mean years ± SD)**	70 ± 16	67 ± 17	73 ± 14	<0.001
**Occlusion site; n (% of all)**				n/a
**- All**	221	17	204	
**- ICA ‘I-type’**	20 (9)	4 (24)	16 (8)	
**- ICA ‘T-type’**	38 (17)	3 (18)	35 (17)	
**- M1**	84 (38)	2 (12)	82 (40)	
**- M2 proximal**	30 (14)	2 (12)	28 (14	
**- M2 distal**	31 (14)	3 (18)	28 (14)	
**- Basilary artery**	10 (5)	1 (6)	9 (4)	
**- Other**	8 (4)	2 (12)	6 (3)	

CTA= computed tomography angiography; EVT= endovascular thrombectomy; Sig.= statistical significance; SD= standard deviation; n/a= not applicable; ICA= internal carotid artery; M1 = first segment of the middle cerebral artery; M2 = post bifurcation segment of the middle cerebral arteries; P1 = first segment of the posterior cerebral artery; A1 = first segment of the anterior cerebral artery; Other = includes occlusions in the intracranial vertebral arteries (2 cases in the CTA group), P1 (5 cases in the EVT group) and A1 occlusions (1 case in the EVT group).

Slice thickness of the analyzed images varied between 0.5 and 1 mm. All examinations were performed on either of the following CT scanners: *Aquilion ONE* (Canon Medical Systems Corporation); *Somatom Definition Flash/Drive/Go/Edge/Edge +* and *AS+*(Siemens Healthineers); *Ingenuity Core/Core 128* and *IQon* (Philips Healthcare Inc.); *Revolution EVO* and *Optima* (GE Healthcare).

Tube voltage ranged from 80 to 120 kV, pitch from 0.6 to 0.9, collimation was 128 × 0.6, 80 × 0.5, or 64 × 0.6 and rotation time ranging from 0.3 to 0.5 s for the Canon, Philips Healthcare and Siemens Healthineers (*Flash/Drive/Go/Edge)* scanners. We did not have access to the full scan protocols for Siemens Edge+ /AS+ or the GE Healthcare scanners since those exams were performed within our region but not within our local clinic.

### 3.2 Diagnostic accuracy

Measures of the diagnostic accuracy for intracranial LVOs in the anterior circulation only (for which the software is trained) are shown for all patients and for subgroups based on occlusion site in Table 2 and for subgroups based on gender or age in Table 3. Measures of diagnostic ability for all LVOs (including both the anterior and posterior circulation LVOs) are shown in Table 4. The highest sensitivity was seen for M1 occlusions (87 %) and T-type ICA occlusions (84 %). The software had profound difficulties detecting I-type (sensitivity 0 %) and M2 occlusions (sensitivity of 13 % and 0 % for proximal and distal M2 occlusions, respectively). The most common software error were false negatives (23 %), however false positives also occurred (3 %). See Table 5 for a summary of the false positives and the potential cause. The software did not detect any of the LVOs in the posterior circulation. Illustrative examples are shown in Fig. 1. Of the six patients with two separate LVOs, the software only detected one M1-occlusion and hence failed to correctly identify any of the multiple LVOs. For all 60 exams, 30 from each group, that were sent for repeated LVO evaluation by the ^AUTO^Stroke CINA, the results were identical to the first round through the software.

**Table 2:** Measures of the diagnostic ability of CINA® LVO for anterior circulation LVOs.

Location of observation (number of occlusions/ total number of observations)	Sensitivity (95 % CI)	Specificity (95 % CI)	PPV (95 % CI)	NPV (95 % CI)
All (n = 203/407)	0.54 (0.47–0.61)	0.93 (0.89–0.97)	0.89 (0.83–0.94)	0.67 (0.64–0.71)
M1 (n = 84/400)	0.87 (0.78–0.93)	0.97 (0.94–0.98)	0.88 (0.80–0.93)	0.97 (0.94–0.98)
M2 proximal (n = 30/400)	0.13 (0.04–0.31)	1.0 (0.99–1.0)	0.80 (0.32–0.97)	0.93 (0.93–0.94)
M2 distal (n = 31/400)	0.0 (0.0–0.11)	1.0 (0.99–1.0)	n/a	0.92 (0.92–0.92)
ICA; I-type (n = 20/400)	0.00 (0.00–0.17)	1.00 (0.99–1.0)	0.00 (n/a)	0.95 (0.95–0.95)
ICA; T-type (n = 38/400)	0.84 (0.69–0.94)	1.00 (0.99–1.0)	1.00 (n/a)	0.98 (0.97–0.99)

LVO= Large vessel occlusion; CI= confidence interval; ICA= internal carotid artery; M1=first segment of middle cerebral artery; M2 = post bifurcation segments of middle cerebral artery; PPV= positive predictive value; NPV= negative predictive value; n/a= not applicable.

**Table 3:** The diagnostic ability of CINA® LVO for anterior circulation LVOs in subgroups based on gender or age.

Subgroup (number of patients/total number of observations)	Sensitivity (95 % CI)	Specificity (95 % CI)	PPV (95 % CI)	NPV (95 % CI)
Female (n = 203/206)	0.57 (0.47–0.67)	0.92 (0.86–0.97)	0.88 (0.79–0.94)	0.69 (0.64–0.74)
Male (n = 197/201)	0.51 (0.41–0.61)	0.95 (0.87–0.98)	0.91 (0.81–0.96)	0.65 (0.61–0.70)
Age <40 years (n = 26/26)	0.67 (0.22–0.96)	1.0 (0.83–1.0)	1.0 (n/a)	0.91 (0.76–0.97)
Age ≥40 and < 70 years (n = 138/140)	0.60 (0.46–0.72)	0.94 (0.86–0.98)	0.87 (0.74–0.94)	0.77 (0.71–0.82)
Age ≥70 (n = 236/241)	0.51 (0.43–0.60)	0.92 (0.85–0.97)	0.90 (0.82–0.95)	0.58 (0.53–0.62)

LVO= Large vessel occlusion; CI= confidence interval; n/a= not applicable.

**Table 4:** Measures of the diagnostic ability of CINA® LVO for posterior circulation LVOs and for all LVOs (anterior and posterior circulation).

Location of observation (number of occlusions/total number of observations)	Sensitivity (95 % CI)	Specificity (95 % CI)	PPV (95 % CI)	NPV (95 % CI)
All posterior and anterior circulation LVOs (n = 221/409)	0.50 (0.43–0.57)	0.93 (0.88–0.96)	0.89 (0.83–0.94)	0.61 (0.58–0.64)
All posterior LVOs (n = 17/400)	0.0 (0.0–0.2)	1.0 (0.99–1.0)	n/a	0.96 (0.96–0.96)
Basilary artery (n = 10/400)	0.00 (0.00–0.31)	1.00 (0.99–1.00)	n/a	0.98 (0.98–0.98)
P1 (n = 5/400)	0.00 (0–00–0.52)	1.00 (0.99–1.00)	n/a	0.99 0.99–0–99)

LVO= Large vessel occlusion; CI= confidence interval; ICA= internal carotid artery; M1=first segment of middle cerebral artery; M2= post bifurcation segments of middle cerebral artery; A1 = first segment of anterior cerebral artery; P1 = first segment of posterior cerebral artery; n/a= not applicable.

**Table 5:** Summary of false positives and any identified potential cause for the misinterpretation.

	All patients (n = 400)
Number of false positives – n (% of all)All M1M2 proximalM2 distalICA; I-type	13 (3) 10 1 1 1
Potential cause – nNo potential cause identifiedAdjacent aneurysmVessel anatomy	9 2 2

CTA= computed tomography angiography;; ICA= internal carotid artery; M1=first segment of middle cerebral artery; M2 = post bifurcation segments of middle cerebral artery.

## 4 Discussion

We evaluated the LVO detection ability of the CINA®LVO software included in Canons ^AUTO^Stroke software package. The overall specificity was high for LVOs in the anterior circulation (93 %). The overall sensitivity for LVOs in the anterior circulation was 54 % with the highest sensitivity for the M1 segment of the middle cerebral artery (87 %) and T-type internal carotid occlusions (84 %). The sensitivity was low for occlusions in the M2 segment of the middle cerebral artery (13 % and 0 % for proximal and distal M2 occlusions respectively), and the software did not detect any of the i-type ICA occlusions nor any posterior circulation occlusions (sensitivity 0 %, although posterior circulation LVOs are not included in the intended use of the software). All cases were successfully processed by the software with response times of 1–2 min (data not shown).

According to the CINA®LVO software manual [13] the software is intended to diagnose LVOs located in the anterior circulation only, including distal ICA, and M1 and M2 (in our study dichotomized as proximal or distal) segments of the MCA. Even though the software is not intended for LVO detection in the posterior circulation, we evaluated posterior circulation LVOs in our study since they are an important clinical entity [5] which are sometimes overlooked in clinical routine because of the unspecific symptoms associated with their clinical presentation [14]. In this study of 221 LVOs in 400 patients, a total of 8 % (n = 17) of all occlusions were located in the posterior circulation. We do not think this is an amount that should be overlooked if the purpose of the software is rapid detection of LVOs in patients potentially eligible for mechanical thrombectomy.

The high specificity of 93 % for the anterior circulation was similar to that shown in previous studies [10], [11], [12]. For the few observed false positives by the CINA® LVO software, a visible reason for the incorrect assessment could, in most cases, be deducted from the images included in the software’s report (for example two cases of partly thrombosed aneurysms and one case with a sharp M2 vessel curve marked as occlusions).

In the study by Rava et al. [11], the overall LVO detection sensitivity and specificity was found to be 73 % and 98 %, respectively. The subgroup analysis, however, revealed an uneven diagnostic ability depending on occlusion site: M2 occlusion sensitivity was 51 % compared to 90 % for the ICA occlusions. McClouth et al. [10] found a sensitivity and specificity for overall LVO detection (including ICA, M1 and M2) to be 98 % and 98 %, respectively. In a third study, by Schlossman et al. [12], the analysis also indicated an uneven performance with a sensitivity of 55 % for LVO detection in the ICA and 87 % for M1 occlusions.

The results of the present study are not entirely in line with the results of neither of these previous studies, we also found the specificity of the software to be high, but with considerably lower sensitivity. It is hard to explain why the sensitivity is lower in our study. One possible reason could be substandard CTA quality, but the software has specific compatibility requirements regarding image parameters/format and if the images do not comply, they will not be evaluated [13]. Since there were no such cases in the study, nor any examinations rated as non-diagnostic by the neuroradiologists, this does not seem like a plausible explanation.

The occlusion site with the lowest sensitivity within the anterior circulation was type-I ICA, i.e. intracranial occlusions of the distal ICA (distal of the ophthalmic artery), sparing the distal ICA terminus. Here, the software did not identify any of the 20 type-I ICA cases included in this study. These occlusions should not be confused with extracranial ICA occlusions, which were not included in our material. A reason for this may be that not enough such occlusions were included in the training data during development of the algorithm, however the software manual states that the software is intended for detection of distal ICA occlusions [13].

The low sensitivity for detection of M2 occlusions, is seen both in our study and in the study by Rava et al. [11]. However, in the Rava et al. study the sensitivity for M2 occlusions was 51 %, but in our study it was much lower with a sensitivity of 13 % for proximal- and 0 % for distal M2-occlusions. Rava et al. included only proximal M2 occlusions in their analysis, while we included both proximal and distal occlusions, evaluating them separately. Therefore, the discrepancy in results between the two studies could not be explained by a categorization difference. A potential reason for this divergence may be differences in CTA protocols, although our material included examinations from several hospitals within a large region. The majority of M2 occlusions included in our study were subjected to mechanical thrombectomy and therefore highly relevant to identify rapidly. The large difference in performance of the software for M2 occlusions warrants caution if used in clinical routine care and should be studied further.

We found differences in sensitivity depending on the age of the subject, with higher sensitivity in the lower age subgroups (sensitivity of 0.67 (age < 40 years), 0,60 (age ≥40 and <70 years) and 0.51 (age ≥70 years). Even though the subgroup with patients under 40 years of age (n = 26) was rather small, so we cannot draw any conclusions based on this, we do believe it warrants further investigation in future studies, especially considering the generally higher age of the target patient group.

There are several LVO detection AI software available. Viz version 4.1.3 software has been found to have a LVO detection sensitivity of 81–90 % and specificity of 82–96 % in consecutive patients examined with CTA [15], and in acute stroke patients [16], respectively. Another available LVO detection software is Rapid CTA, which has been shown to have a sensitivity of 94–96 % and a specificity of 76–98 % [17], [18] for ICA and M1 occlusions. Most of these AI platforms also perform intracerebral hemorrhage detection and perfusion analysis as a complete AI evaluation solution for acute stroke patients [9]. Potentially, the test performance, specifically the sensitivity, could be further improved by using the results of the perfusion study as an aid in the assessment of LVOs by combining the perfusion and LVO results to make the software more sensitive to LVOs if there is a perfusion defect.

### 4.1 Strengths and limitations

Strengths of this study include the consecutive patient material and that the LVO population was enriched by evaluating patients who had been treated with endovascular thrombectomy. There are limitations to this study, primarily that we only evaluated one LVO detection software. Another limitation is that we considered the original radiology report, evaluated by at least one neuroradiologist, as “ground truth” and did not reevaluate the exams systematically for the purpose of this study unless the results differed between the software and the report. Conventional angiography might have been the most correct ground truth since severe stenosis in some cases could be evaluated as an occlusion on CTA, however, not all patients in the target group for LVO-detection software will be subject to conventional angiography and therefore CTA evaluation by a neuroradiologist was considered ground truth.

### 4.2 Conclusion

We confirm that the CINA ®LVO software for LVO detection has high sensitivity and specificity for ICA T-occlusions and MCA M1 occlusions, but the sensitivity is low for intracranial distal ICA type-I occlusions and for proximal as well as distal M2 occlusions, which is contrary to previous reports and warrants caution if used in clinical routine care and should be studied further. In order to be an effective triage tool, addition of posterior circulation LVO detection ability is warranted.

Further studies evaluating multiple supplier’s software side-by-side in population-wide datasets would be of value, as would studies of the clinical workflow to evaluate time-to-diagnosis to illustrate the effect of LVO detection software in clinical routine care.

## Funding

This study has received no specific funding but was supported by the 10.13039/501100003173Crafoord Foundation (#20200548), 10.13039/501100011077Skåne University Hospital (#96437, #96438) and 10.13039/501100009780Region Skåne (YF-ALF 43435) to JW and Region Skåne (ST-ALF 47740) to HM. The funders had no active role in the methodology, data collection/analysis or writing of the manuscript.

## Ethical statement

Written informed consent was not required for this study because of the retrospective method and that no interventions altering the course of the patients’ workup or treatment were performed, the CT images in the figures are entirely anonymised images and the individuals cannot be identified.

Institutional Review Board approval was not required for the same reasons stated above.

## CRediT authorship contribution statement

**Helena Mellander:** Data curation; Formal analysis; Funding acquisition; Investigation; Methodology; Project administration; Visualization; Software; Writing - original draft. **Amir Hilal:** Data curation; Formal analysis; Investigation; Methodology; Project administration; Software; Validation; Writing - review & editing. **Teresa Ullberg:** Conceptualization; Methodology; Supervision; Validation; Writing - review & editing. **Johan Wassélius:** Conceptualization; Data curation; Funding acquisition; Methodology; Project administration; Resources; Validation; Software; Writing - review & editing.

## Declaration of Competing Interest

The authors declare that they have no known competing financial interests or personal relationships that could have appeared to influence the work reported in this paper.

## Data Availability

Requests to access an anonymized dataset supporting the conclusions of this article may be sent to the corresponding author.
