# Supplementary material for: Evaluation of CINA® LVO artificial intelligence software for detection of large vessel occlusion in brain CT angiography
Source: Eur J Radiol Open. 2023 Dec 15;12:100542. doi: 10.1016/j.ejro.2023.100542 (PMC10764253; doi:10.1016/j.ejro.2023.100542)
Supplement: Supplementary material [file mmc1.docx]

|  | CTA patients (n=200) |
| --- | --- |
| Urgency of exam – n (%)   - Acute (< 24 hours) - Sub-acute (< 1 week) - Non acute | 169 24 7 |
| Indication for exam – n (%)   - Fast track ‘stroke’ - Persistent neurol. deficits - Transient neurol. deficits - Headache - Confusion - Trauma - Other or multiple | 54 37 36 14 5 5 48 |

**Supplemental table 1.** Summary of urgency of exam and indication for exam for the patients in the CTA group.

**CTA**= computed tomography angiography; **Indication for exam ‘Other’**= includes for example patients with established ischemic lesions going through further work up, vertigo, loss of consciousness, planned follow up of known stenosis or aneurysms.
